# Robustness Assessment of Images From a 0.35T Scanner of an Integrated MRI-Linac: Characterization of Radiomics Features in Phantom and Patient Data

**DOI:** 10.1177/15330338221099113

**Published:** 2022-05-06

**Authors:** Rebecka Ericsson-Szecsenyi, Geoffrey Zhang, Gage Redler, Vladimir Feygelman, Stephen Rosenberg, Kujtim Latifi, Crister Ceberg, Eduardo G. Moros

**Affiliations:** 1 Department of Medical Radiation Physics, Clinical Sciences, 5193 Lund University, Lund, Sweden; 2 Radiation Oncology Department, 25301 H. Lee Moffitt Cancer Center and Research Institute, Tampa, FL, USA

**Keywords:** cancer, MRI, prediction, quantification, radiation therapy, validation, biomarker

## Abstract

**Purpose:** Radiomics entails the extraction of quantitative imaging biomarkers (or radiomics features) hypothesized to provide additional pathophysiological and/or clinical information compared to qualitative visual observation and interpretation. This retrospective study explores the variability of radiomics features extracted from images acquired with the 0.35 T scanner of an integrated MRI-Linac. We hypothesized we would be able to identify features with high repeatability and reproducibility over various imaging conditions using phantom and patient imaging studies. We also compared findings from the literature relevant to our results. **Methods:** Eleven scans of a Magphan^®^ RT phantom over 13 months and 11 scans of a ViewRay Daily QA phantom over 11 days constituted the phantom data. Patient datasets included 50 images from ten anonymized stereotactic body radiation therapy (SBRT) pancreatic cancer patients (50 Gy in 5 fractions). A True Fast Imaging with Steady-State Free Precession (TRUFI) pulse sequence was selected, using a voxel resolution of 1.5 mm × 1.5 mm × 1.5 mm and 1.5 mm × 1.5 mm × 3.0 mm for phantom and patient data, respectively. A total of 1087 shape-based, first, second, and higher order features were extracted followed by robustness analysis. Robustness was assessed with the Coefficient of Variation (CoV < 5%). **Results:** We identified 130 robust features across the datasets. Robust features were found within each category, except for 2 second-order sub-groups, namely, Gray Level Size Zone Matrix (GLSZM) and Neighborhood Gray Tone Difference Matrix (NGTDM). Additionally, several robust features agreed with findings from other stability assessments or predictive performance studies in the literature. **Conclusion:** We verified the stability of the 0.35 T scanner of an integrated MRI-Linac for longitudinal radiomics phantom studies and identified robust features over various imaging conditions. We conclude that phantom measurements can be used to identify robust radiomics features. More stability assessment research is warranted.

## Introduction

### Background

Image-guided radiation therapy (IGRT) has experienced considerable advancements since the development and implementation of onboard cone-beam computed tomography (CBCT) systems.^1^ Recently, radiation therapy systems with integrated MRI scanners have been introduced clinically providing superior soft-tissue contrast compared to X-ray-based imaging.^2^ In addition to RECIST and other similar protocols based on visible tumor measurements [https://recist.eortc.org/], computed tomography (CT), positron emission tomography (PET), and magnetic resonance imaging (MRI) images are qualitatively analyzed by radiologists as a standard practice for screening, staging or decision-making purposes.^3^ Quantitative analysis, or radiomics, aims to extract additional information from these standards of care images with the hypothesis that texture and voxel value distribution contain physiological information not discernable visually.^4^ Images are converted into mineable data generating so-called radiomics features (imaging biomarkers) relating to pathophysiological processes which, combined with other patient data, are hypothesized to provide predictive or discriminative information.^4-6^ By combining qualitative and quantitative data, the long-term goal is to build reliable descriptive clinical models, tailoring treatment to each patient and provide even further personalized oncology than available today.^7-9^

Quantitative image analysis can be divided into several steps: image acquisition, segmentation, feature extraction, statistical analysis, and model building, each with unique challenges.^5,8,10^ Features can be vulnerable to differences between and within image modalities such as fundamental imaging physics, imaging parameters, reconstruction methods, a segmentation method, feature extraction software, etc.^8-12^ Comparison between institutions is therefore difficult and the lack of standardized methodologies is a major challenge for radiomics to overcome before clinical translation.^5,8,9^ Furthermore, models based on nonrobust features will likely not provide reliable predictions when applied prospectively to new data.^13^ Although no standardized guidelines on how to assess feature robustness have been developed, it is emphasized by The Image Biomarker Standardization Initiative (IBSI)^6^ as a primary step in the feature selection process.^14^ IBSI is an independent international collaboration aiming to establish common biomarker nomenclature and definitions for the radiomics community. Thus, identifying features that are robust under various imaging conditions is essential to develop clinical outcome prediction or clinical decision support systems.^5,14^

ViewRay's MRIdian MRI-Linac (ViewRay Inc., Cleveland, OH) is a commercially available hybrid system combining a 0.35T scanner with a 6 MV flattening-filter-free (FFF) medical electron linear accelerator.^15^ This system provides a potentially advantageous setting in which images for radiomics analysis are acquired within the context of radiotherapy treatment on a daily basis. However, reliable approaches and robust radiomics features acquired with this MRI-guided radiotherapy (MRIgRT) workflow still remain to be determined. In this retrospective study, we investigated radiomics features in both phantom and patient images acquired with the scanner of such a system, with a primary focus on robustness assessment, investigating the repeatability and reproducibility of the system and associated radiomics feature calculations. The aim was to explore longitudinal radiomics studies in invariant objects as well as identifying robust radiomics features across various imaging conditions. Additionally, a literature review over MRI-based radiomics with emphasis on either assessing robustness or various clinical correlations was included in this work for comparison, and to identify potential features fulfilling both the robustness and predictive criteria.

### Literature Review

The main aim of this work was to investigate feature variability and performing a robustness assessment of the integrated MRI-Linac system in both phantom and patient data. A literature review with the purpose of providing a comprehensive summary of other available similar studies within MRI-based radiomics was included. The main literature collection took place between January and May 2020, but a few later published papers have been included after this. Most literature^14,16-28^ was found through the PubMed database searching for, for example, “MRI radiomics,” “MRI Linac radiomics,” “MRI radiomics stability,” “Radiomics phantom study,” etc. A summary of published studies with similar questions, aims, or other relevant findings regarding feature variability based on their relevance to our study were therefore included. The primary goal was to characterize robust features in various imaging conditions. It is important to recall that feature robustness is not an implication of feature predictability or other biomarker correlation to any clinical task or outcome.^14^ A secondary goal of the literature review was therefore to identify common radiomics features demonstrating both high robustness and significant clinical correlation. Thus, a summary of the relevant papers included in the literature review can be seen in Tables 1 and 2, where the study purpose, feature classes, and robust/predictive features consistent with the findings in this work are presented.

**Table 1.** Summary of MRI-Based Radiomics Robustness Assessment Papers.

Title	Author	Study purpose	Scanning system	Feature classes	Statistical measure	Common features
Robustness of radiomic features in magnetic resonance imaging: review and a phantom study.	Cattell et al^14^	Explore feature variability due to variations in SNR, ROI delineation, small voxel size variation, and normalization method.	3T	First order, shape-based, GLCM and GLRLM	ICC*	Sphericity and Spherical disproportion (shape); Inverse difference and Sum entropy (GLCM); SRE, RPC, LRE, and RLNU (GLRLM)
Stability and variability of radiomics features on a 0.35 T MR-guided-RT system.	Padgett and Mihaylov^16^	Feature variability study using phantom measurements.	0.35 T integrated MRI-Linac	Shape-based, first order and GLCM	CoV	Surface area, surface-to-volume ratio, compactness 1 and spherical disproportion (Geometric); Hist entropy (First ord.); Entropy (GLCM)
Lack of robustness of textural measures obtained from 3D brain tumor MRIs impose a need for standardization.	Molina et al^17^	Investigate effects on feature variability when altering dynamic range and spatial resolution.	3T	Second order (GLCM and GLRLM)	CoV	GLCM entropy
Multicenter evaluation of MRI-based radiomic features: A phantom study.	Rai et al^18^	Explore reproducibility between scanners, using a novel 3D-printed radiomics phantom	1.5 T–3 T	Shape-based, first order and second order	CoV (intrascanner variability); ICC* (interscanner variability)	Intra- and interscanner: entropy and sum entropy (GLCM); SRE, LRE, RLNU, and RPC (GLRLM)
Quantitative variations in texture analysis feature dependent on MRI scanning parameters: A phantom model.	Buch et al^19^	Look at feature variability when varying magnet strength, flip-angle, NEX, and scanner platform.	1.5 T–3 T	Histogram, GLCM, GLRLM, GLGM, and Laws	Two-tailed *t*-test	None
Extracting and selecting robust radiomic features from PET/MR images in nasopharyngeal carcinoma.	Yang et al^20^	Explore feature variability and redundancy in patients with nasopharyngeal carcinoma (NPC).	3 T	Intensity, textural	ICC*	Entropy (GLCM) and entropy (HLH)
Repeatability of radiomic features in magnetic resonance imaging of glioblastoma: test–retest and image registration analyses.	Shiri et al^21^	Stability assessment of features in glioblastoma tumors using different registrations and field inhomogeneity corrections.	1.5 T	Shape-based, first order, textural	ICC*	Entropy (first order); entropy (GLCM) and energy (Wavelet LLL)
Delta radiomics analysis of magnetic resonance-guided radiotherapy imaging data can enable treatment response prediction in pancreatic cancer.	Tomaszewski et al^22^	Investigating the effects of image intensity normalization and spatial robustness analysis before treatment response prediction.	0.35 T integrated MRI-Linac	Histogram, GLCM, GLRLM, GLSZM, and NGTDM.	CCC**	RLNU, RPC, SRE, and LRE (GLRLM); inverse difference moment and inverse difference (GLCM)

**Table 2.** Summary of MRI-Based Radiomics Looking at Various Clinical Correlations.

Title	Author	Study purpose	Scanning system	Feature classes	Common features
Delta radiomics for rectal cancer response prediction with hybrid 0.35 T magnetic resonance-guided radiotherapy (MGRT): a hypothesis-generating study for an innovative personalized medicine approach.	Boldrini et al^23^	Study predictive performance of delta radiomics in rectal cancer patients.	0.35 T integrated MRI-Linac	Shape-based, statistical, fractal and GRLRM	Volume, sphericity, asphericity, compactness 1, spherical disproportion (shape); SRE, LRE, RLNU, RPC (GLRLM)
MRI radiomic features are independently associated with overall survival in soft tissue sarcoma.	Spraker et al^24^	Look at the association between radiomic features and overall survival in patients with soft tissue sarcoma.	0.7 T, 1.5 T, and 3T	Tumor volume, intensity histogram, GLCM, NGTDM, and GLSZM	Volume (shape), Hist entropy, entropy and inverse difference moment (GLCM)
Correction for magnetic field inhomogeneities and normalization of voxel values are needed to better reveal the potential of MR radiomic features in lung cancer.	Lacroix et al^25^	Explore how preprossesing affects predictive performance.	3 T	Shape-based, first and second order	Volume (shape); entropy (GLCM); SRE, LRE, and RLNU (GLRLM)
Predictive value of 0.35 T magnetic resonance imaging radiomic features in stereotactic ablative body radiotherapy of pancreatic cancer: A pilot study	Simpson et al^26^	Study predictive performance for features from pancreatic cancer patients.	0.35 T integrated MRI-Linac	First and second order	Entropy (GLCM)
Computer-aided diagnosis of breast DCE-MRI using pharmacokinetic model and 3D morphology analysis prediction in breast MRI.	Wang et al^27^	Characterize breast lesions using a computer-assisted algorithm.	1.5 T	Shape-based and GLCM	Entropy, inverse difference moment, and Sum entropy (GLCM)
Central gland and peripheral zone prostate tumors have significantly different quantitative imaging signatures on 3 Tesla endorectal, in vivo T2-weighted MR imagery.	Viswanath et al^28^	Evaluate textural features in prostate cancer patients.	3 T	Texture	Entropy, inverse difference moment, and sum entropy (GLCM)
Delta radiomics analysis of Magnetic Resonance guided radiotherapy imaging data can enable treatment response prediction in pancreatic cancer.	Tomaszewski et al^22^	Exploring delta radiomics performance for treatment response prediction in pancreatic cancer patients.	0.35 T integrated MRI-Linac	Histogram, GLCM, GLRLM, GLSZM, and NGTDM.	None

## Materials and Methods

### Phantom Properties

**Figure 1.**
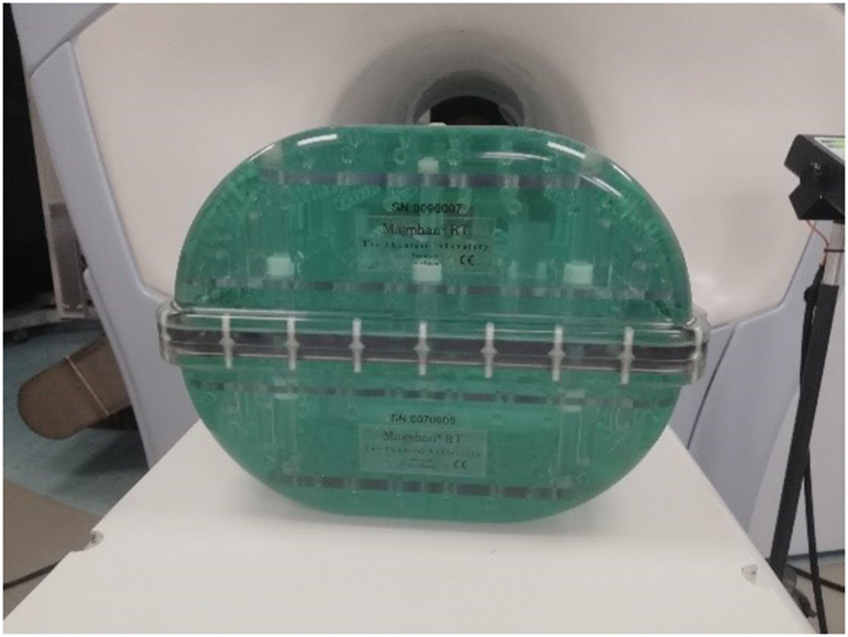
Magphan^®^ RT phantom.

The Magphan^®^RT Phantom (Figure 1) consists of 2 parts: a top module (TMR009) and a bottom module (TMR007). Both contain >100 spherical fiducials and other solid test components filled with an MRI-signal generating liquid (96.4% distilled water, 2.5% PVP, 0.9% sodium chloride, <0.2% potassium sorbate, <0.2% copper sulfate, and <0.2% blue food color, all defined in percentage by weight). This results in T1 and T2 values of about 175 to 225 ms at 0.35 T.^29,30^

**Figure 2.**
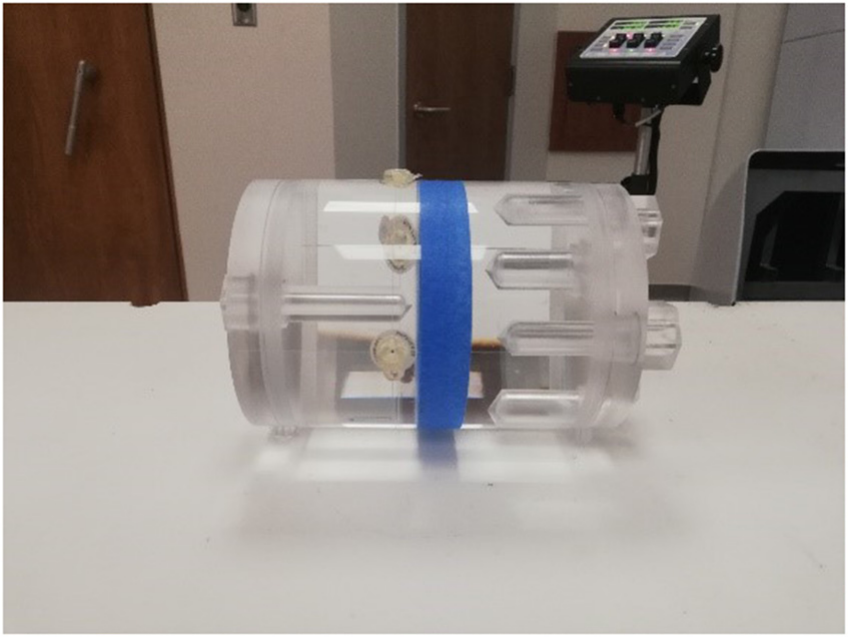
ViewRay Daily QA phantom.

The ViewRay Daily QA Phantom (Figure 2) is a cylindrical phantom filled with distilled water. It has 1 central and 4 surrounding cavities for insertion of an ionization chamber.^31^

### Data Selection

Eleven scans acquired over a 13-month period using the Magphan^®^RT Phantom, and acquired over 11 workdays using the ViewRay Daily QA Phantom, respectively, constituted the complete phantom dataset.

The Institutional Review Board at the University of South Florida approved (IRB #20383) and waived the informed consent requirement for retrospective analysis in this study. Patient data included 50 images from 10 anonymized stereotactic body radiation therapy (SBRT) pancreas cancer patients treated with 50 Gy in 5 consecutive daily fractions. The kidneys and liver were chosen to represent theoretically invariant objects in the patient, assuming no significant effect of radiation during the course of treatment and consistent distance/orientation relative to the pancreatic target, thus ensuring consistent location within the imaging coils. Both organs exhibit a desirable heterogeneity for radiomics studies, thus being appropriate alternatives as a transition from ideal imaging conditions to more complex structures as human tissue.

In summary, 4 datasets were included for statistical analysis of calculated radiomics features defined as follows: monthly phantom, daily phantom, patient kidney, and patient liver.

### Image Acquisition and Registration

All phantom images were acquired using a torso coil and high-resolution TRUFI pulse sequence with imaging parameters: 1.5 mm × 1.5 mm × 1.5 mm resolution, 500 mm × 449 mm × 432 mm Field of View (FOV) and 172 s total image acquisition time. Positioning and set-up were identical for every scanning occasion. All patient images were acquired using a torso coil and TRUFI pulse sequence with 1.5 mm × 1.5 mm × 3.0 mm resolution, 540 mm × 465 mm × 432 mm FOV and 25 s total imaging time (for faster imaging during treatment). Image export, import, segmentation, and registration were done in Mirada RTx (Mirada RTx 1.6, Mirada Medical, Oxford, UK).

**Figure 3.**
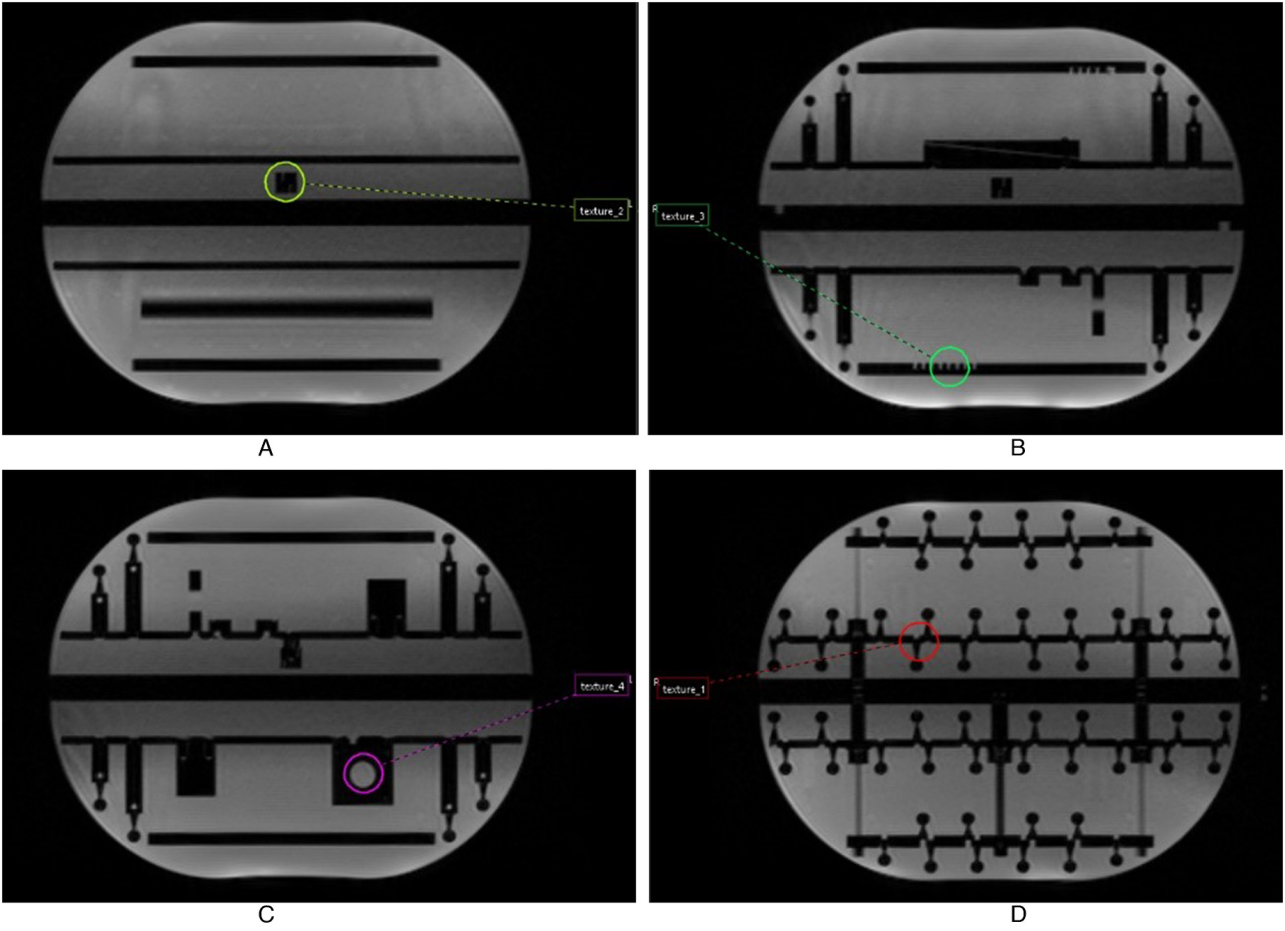
(a)-(d) Four cylindrical VOI were placed in various regions in the Magphan^®^ RT phantom displaying heterogeneous patterns.

**Figure 4.**
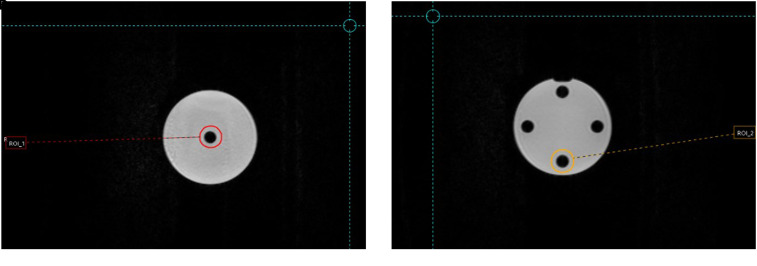
(a), (b) Two VOI of the same size were placed in the ViewRay Daily QA phantom.

**Figure 5.**
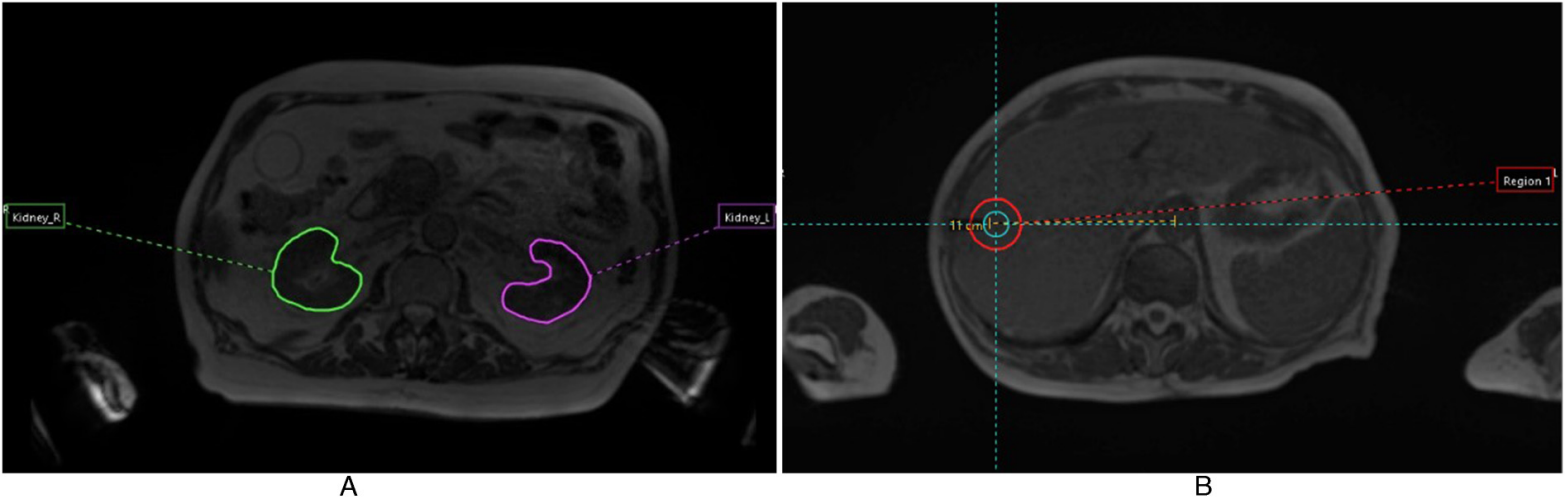
(a) Kidneys were manually segmented for each patient image, (b) a spherical VOI was placed in the liver for each patient and scanning occasion.

Identical cylindrical 4.2 cm^3^ VOIs were contoured in different sections of both phantoms: 4 regions in the Magphan^®^RT Phantom (Figure 3) and 2 regions in the ViewRay Daily QA Phantom (Figure 4). All structures were propagated from the baseline to the remaining ten imaging sets by rigid registration in Mirada RTx. For each patient image a spherical 14 cm^3^ VOI was placed in the midsection anteriorly/posteriorly, 4 cm caudally from the diaphragm, and 11 cm laterally from the aorta (Figure 5b), while kidneys were manually segmented by a single user (Figure 5a).

### Statistical Analysis

Traverso et al^3^ defined feature robustness into 2 main elements: repeatability and reproducibility. Repeatability refers to the agreement between measurements under identical imaging conditions, that is, intrasubject scanning using identical scanning parameters, set-up, equipment, etc. Reproducibility refers to the degree to which features stay unchanged under various imaging conditions, for example, identical imaging parameters but different subjects, different imaging parameters but the same subject, etc. In this study, features fulfilling both of these requirements were classified as robust.

In this work, the CoV was chosen as the figure of merit for robustness quantification since it allowed for a straightforward methodology to identify robust features within and between many subjects. It is defined as

$$\mathrm{CoV}=100\cdot \;\frac{\sigma }{\left|\mu \right|}$$

where s is the standard deviation and |µ| is the absolute value of the mean. CoV describes the dispersion of the data points, expressed as a percentage, where low values indicate high stability and vice versa.

### Feature Extraction and Statistical Workflow

An in-house program, whose definitions are based on IBSI recommendations and those found in the work by Shafiq-ul-Hassan et al,^9^ was used to extract 1085 shape-based, first, second, and higher-order features (Table 3). Shape-based features describe various geometric properties of the VOI, such as volume, compactness, surface area, etc.^6,10^ First order features relate to voxel intensity distribution within the VOI, with no regard to their relative spatial distribution.^5^ Most of these features require intensity discretization of the 2D or 3D data before calculation.^6,10^ Second-order statistics, also referred to as texture features, provide both intensity and spatial information. They describe the distribution of voxel intensity values between neighboring voxels along with different directions and distances and are derived from so-called **gray-tone-spatial-dependence matrices**.^5,6,32^ The matrices used in this work were the gray-level co-occurrence matrix (GLCM), the gray-level run-length matrix (GLRLM), the gray-level size zone matrix (GLSZM), and the neighborhood gray-tone difference matrix (NGTDM). A full description of how these matrices are defined and of the subsequent feature extraction based on a 26-connected region in 3D is given in the IBSI manual.^6^ Lastly, the higher-order statistical features apply various noise reduction or detail identifying filters on the images before feature extraction.^5,7^ The filter-based approaches used in this study were Laws’,^33,34^ wavelets,^35-37^ Laplacian transforms of Gaussian-filters (LoG),^5^ and fractal analysis.^5,38^

**Table 3.** Complete Summary of Feature Sub-Categories, Number of Extracted Features are Written Within Parentheses.

Feature category	
Shape-based (35)	Laws SEE (22)
First-order (62)	Laws SEL (22)
Co-occurrence (40)	Laws SES (22)
Run-length (17)	Laws SLE (22)
Gray-level size zone (12)	Laws SLL (22)
Neighborhood gray tone diff. (11)	Laws SLS (22)
Laws EEE (22)	Laws SSE (22)
Laws EEL (22)	Laws SSL (22)
Laws EES (22)	Laws SSS (22)
Laws ELE (22)	Wavelet HHH (22)
Laws ELL (22)	Wavelet HHL (22)
Laws ELS (22)	Wavelet HLH (22)
Laws ESE (22)	Wavelet HLL (22)
Laws ESL (22)	Wavelet LHH (22)
Laws ESS (22)	Wavelet LHL (22)
Laws LEE (22)	Wavelet LLH (22)
Laws LEL (22)	Wavelet LLL (22)
Laws LES (22)	LoG sigma = 0.5 mm (22)
Laws LLE (22)	LoG sigma = 1.0 mm (22)
Laws LLL (22)	LoG sigma = 1.5 mm (22)
Laws LLS (22)	LoG sigma = 2.0 mm (22)
Laws LSE (22)	LoG sigma = 2.5 mm (22)
Laws LSL (22)	LoG sigma = 3.0 mm (22)
Laws LSS (22)	Fractal dimension (6)

Repeatability and reproducibility were assessed with CoV < 5% as the threshold for feature robustness. Feature extraction was carried out for all imaging sessions and VOIs in each patient/phantom, followed by calculation of CoV. For both phantom datasets, each VOI was initially treated separately. The mean value of CoV for all VOIs (4 VOIs in the monthly phantom dataset and 2 in the daily) was then evaluated and robust features (CoV < 5%) in each dataset were identified. A similar initial feature selection procedure was applied to the patient kidney and liver data, respectively, calculating CoV for all features in each individual patient dataset first. Robust features were then identified by looking at the CoV mean between all patients within the kidney and liver datasets separately. Features fulfilling the robustness criteria in all 4 datasets were selected in the final step. Thus, the statistical workflow took into account both the repeatability and reproducibility criteria by looking at intrasubject variability in the first step, followed by intersubject analysis between different patients and as well in the final feature selection process.

## Results

In this study 130 out of 1085 radiomics features demonstrated high robustness in both phantom and patient data. Robust features were identified in every category apart from GLSZM and NGTDM. All final robust features (CoV < 5%) are presented in Table 4 and quantitative results are presented in Appendix (Table 6). Out of the 130 features that we identified, 17 were characterized as robust in the literature review while 13 features were found to have significant discriminative or predictive power in various clinical tasks mentioned in the literature (see Table 5). Features found to be both robust and predictive in the literature are marked in bold. The textural feature **GLCM** **entropy** is noticeable in several papers mentioned in Tables 1 and 2, and furthermore is highlighted in more studies^39,40-42^ as a significant differentiator between malignancies and noncancerous tissue in breast and prostate MRI scans. This particular feature has high robustness in our work and in 3 other stability assessment studies.

**Table 4.** Selected Robust Features (CoV < 5%) in Both Phantom and Patient Data Sorted by Sub-Category.

Shape-based	First order	GLCM	GLRLM	LoG sigma = 0.5 mm	LoG sigma = 1 mm	LoG sigma = 1.5-3 mm	Fractal dimension	Wavelet LLL, HHH	Wavelet LLH	Wavelet LHL, HLL, LHH, HLH	Wavelet HHL	All Laws categories (apart from LLL)	Laws LLL
V (voxels)	Volume fraction at 0.10 intensity	Entropy	Short-run emphasis	Energy	Entropy	Coeff vari	MeanLac1	Coeff vari	Coeff vari	Entropy	Entropy	Hist entropy	Energy
Volume	NIenergy	Mean	Long-run emphasis	Entropy	Hist entropy	Energy	MeanLac2	Energy	Entropy	Hist entropy	Hist entropy		Entropy
Surface area	Entropy	Inverse diff. moment	Run length non-uniformity	Hist entropy	Norm entropy	Entropy	MeanLac3	Entropy	Hist entropy	Norm entropy			Hist entropy
Surface-to-volume ratio	Hist entropy	Inverse difference	Run percentage	Norm energy		Hist entropy		Hist entropy	Norm entropy				Norm energy
Volume density (axis)	Norm NIenergy	Sum entropy		Norm entropy		Norm energy		Norm energy					Norm entropy
Area density (axis)	Norm entropy	Vnorm mean				Norm entropy		Norm entropy					
Volume density (convex)		Gnorm entropy											
Area density (convex)		Gnorm sum entropy											
Sphericity		Gnorm mean											
Asphericity		Vgnorm Mean											
Compactness 1													
Spherical disproportion													
Long axis (mm, COM)													
Maximum 3D diameter (mm)													

**Table 5.** Robust Features Fulfilling the Robustness or Predictive Criteria in the Chosen Literature Review That are Common to Our Result. Features Marked in Bold are Found to be Both Robust and Predictive in the Literature.

Robust features	Predictive features
**Compactness 1**, surface area, surface-to-volume ratio, sphericity & **spherical disproportion** (shape), entropy & Hist entropy (first order), **entropy, sum entropy, inverse difference moment** & inverse difference (GLCM), entropy (HLH), **SRE, RPC, LRE, RLNU** (GLRLM), energy (wavelet LLL)	Volume, sphericity, asphericity, **compactness 1, spherical disproportion** (shape), **entropy**, Hist entropy, **sum entropy** & **inverse difference moment** (GLCM), **SRE, LRE, RLNU, & RPC** (GLRLM)

Note: The code to compute radiomic features used in this paper can be shared upon request.

## Discussion

Our literature review included both phantom and patient data analysis, as well as different approaches to investigate reproducibility and repeatability. Cattell et al^14^ performed phantom measurements looking at variability due to altering signal-to-noise ratio (SNR), ROI delineation, small voxel size variation, and normalization methods. They concluded that many features are nonrobust over these variations. The work by Rai et al^18^ used a 3D-printed phantom for exploring intra- and inter-scanner variability. They identified robust first order and texture features but also reported an overall noticeable variation in feature robustness. The phantom study by Buch et al^19^ looked at the effect of varying magnet strength, flip-angle, number of excitations (NEX), and scanner platform and found no features that were stable across all alterations. Although results varied among peer-reviewed papers, most authors agreed that many features are sensitive to several external factors and that further research in order to understand their behavior is essential.

Several studies explored novel methods of designing MRI texture radiomics phantoms. Rai et al^18^ designed a 3D-printed phantom and Buch et al^19^ constructed a phantom using doped gel-filled tubes. Valladares et al^43^ presented a summary of various MRI texture phantom analysis studies in which different materials for simulating tumor heterogeneity were used. Most designs consisted of solid structures, usually polystyrene spheres or porous foams embedded in an agarose gel mixture. However, limitations regarding sensitivity to temperature and humidity are 2 factors to be overcome before handling these in multicenter trials. The phantoms in our work were designed for QA and consisted of homogeneous structures giving rise to a close to a binary signal. Prospective research would be to expand our analysis to texture phantoms similar to those found in the literature mentioned.

Radiomics is a fast emerging area and several studies on the subject have therefore been published since the time of our literature review. Sun et al^44^ presented a recent phantom study on robustness analysis of images from a 1.5 T scanner of an integrated MRI-Linac. Like our results, they found a significant effect on feature variability from the test–retest cohort and therefore emphasize the importance of removing features that are sensitive to machine influence. No common robust features were identified between their work and ours. In another phantom study by Wong et al,^45^ they investigated longitudinal feature repeatability on two 1.5 T scanners by acquiring 30 consecutive daily images of an ACR MRI phantom. Five of their repeatable shape-based features overlapped with our results, namely: maximum 3D diameter, sphericity, surface area, surface-to-volume ratio, and voxel volume. It should be noted that **Maximum 3D diameter** and **Voxel volume** were not identified in our literature review. Xue et al^46^ investigated feature repeatability, reproducibility, and within-subject agreement in a clinical environment, looking at prostate cancer patients scanned on both a 1.5 T MRI-simulator and a 1.5 T MRI-Linac. Two robust features overlapped with our study: energy (wavelet LLL) and run-length nonuniformity (GLRLM). The authors conclude that a significantly smaller proportion of features pass the robustness criteria in their study, compared to a phantom study on the same MRI scanner and protocol. In agreement with our conclusions, they also emphasize the wider range of heterogeneity in patient data compared to phantoms.

We used an in-house developed program, based on the definitions given by IBSI, for feature extraction. However, studies show that features might be vulnerable to the choice of extraction software since calculation settings can vary.^12,47^ Fornacon-Wood et al^47^ compared the outcome between 4 platforms, 3 of which were IBSI-compliant, and concluded that choice of the program has an effect on feature variability as well as their correlation to clinical outcome. In the work by McNitt-Gray et al,^12^ they looked at the agreement between different radiomics software packages under controlled conditions using standardized radiomics feature definitions (using the IBSI manual). They concluded that high levels of agreement between packages were achieved for some of the features while feature definitions requiring more complex derivations did not show the same levels of agreement. Thus, although standard definitions are being used, the choice of feature extraction software has an impact on the final determination, which should be taken into consideration when analyzing and comparing results. There is progress towards reaching common ground, but variations are still prevalent and remain a challenge for radiomics studies.

Another limitation to our analysis lies in the choice of cylindrical and spherical VOIs for phantom and patient (liver) data, respectively. These shapes do not have any unique long or short axis, which is of relevance for calculating many of the shape-based features. Volume and area are not affected but it is worth considering that some shape-based features may lose their meaning in these datasets.

Gray-level normalization is recommended^11,20,21,25^ before feature extraction and analysis to reduce the effects of using different scanners, protocols, and reconstruction parameters. As concluded by Lacroix et al^25^ image processing correcting for, for example, magnetic field inhomogeneity or voxel value normalization are 2 of numerous aspects shown to affect feature outcome. The effect of gray-level normalization is further emphasized by Collewet et al.^11^ In our study each dataset was acquired with the same scanner and protocol. Since each dataset was analyzed separately before identifying common robust features among all data, normalization was omitted as it was assumed that the system produced similar images under the same imaging conditions. In fact, our robustness analysis is temporal to discern the potential effects of scanner drift on feature robustness. Interestingly, a recent study on a similar 0.35 T MRI-Linac system by Tomaszewski et al^22^ looked at treatment response prediction for delta radiomics in pancreatic cancer patients and concluded that normalization reduces interscan signal variations as well as nonpathologic signal drift. They emphasize the importance of image preprocessing and robustness analysis before feature selection and present an explicit normalization method. We acknowledge that there may be many preprocessing techniques to improve feature robustness (SNR). Our assumption of no scanner drift is therefore a more conservative approach for the selection of robust features.

Our results indicate that 13 radiomics features overlapped between our analysis and with those identified as predictive/prognostic in the literature review. Boldrini et al^23^ looked at a similar 0.35 T MRI-Linac system as in this work whereof 9 common features could be identified. Although preliminary, this is a promising result suggesting a useful potential for radiomics studies on such a system across scanners and institutions. In another study on the same system by Tomaszewski et al,^22^ several common features were identified in their robustness analysis, but no overlap was seen between their predictive features and our results; this can be expected since the test for robustness was completely different. The textural feature GLCM entropy has been characterized as a significant classifier for lesion discrimination in several studies as well as in stability assessment papers. The results are promising by identifying radiomics features for further investigation. Although a large number of features were classified as robust in our work, a substantial proportion were not (88%). MRI-based radiomics stability assessment has been investigated but to a limited extent, thus even though efforts are made in finding common methods, no consensus in stating feature robustness or their predictive power currently exists. The situation where features are found to be predictive but not robust must be further investigated. We, therefore, stress the importance of reporting feature variability and further emphasize the relevance of robustness assessment as a first step before starting any useful clinical correlation.

This work has investigated the robustness assessment of a 0.35 T integrated MRI-Linac with respect to derived radiomics features and provides a comprehensive and novel summary of longitudinal radiomics on such a system. We identified 130 robust features and conclude that certain radiomics features on images acquired with the low-field scanner of the system are stable over time. Phantom and human data were analyzed separately as a prior step, while the final analysis entailed a joint comparison and extraction of common robust features, which to our knowledge has not been performed on such a system before. Although no texture phantoms were used that reflect the complexity and wide range of gray levels observed in human tissue, the phantom analysis is valuable for representing ideal imaging conditions in a controlled experimental setting. Combined with patient data it is therefore useful as an indication of variability solely due to inherent machine properties. Thus, it is in our future interest to develop a heterogeneous phantom to further explore and confirm feature behavior on a low-field MRI-Linac.

## Conclusion

This work has explored the longitudinal robustness of radiomics features studies on a low-field integrated MRI-Linac and assessed that the 0.35 T scanner of the system is sufficiently stable over time for such analysis. Our results indicate that robust features over a wide range of imaging conditions can be identified in both phantom and patient data, and we emphasize the usefulness of phantom studies for feature stability assessment as it provides a controlled setting. Developing a functional texture phantom for MRI-based radiomics would be of great interest in future studies. Furthermore, a literature review revealed that several of the features demonstrating a high level of stability in our analyses have also been found to be significantly related to various clinically relevant factors.

## Abbreviations

MRI, magnetic resonance imaging; SBRT, stereotactic body radiation therapy; TRUFI, true fast imaging with steady state free precession; CoV, coefficient of variation; GLSZM, gray level size zone matrix; NGTDM, neighborhood gray tone difference matrix; IGRT, image guided radiation therapy; CBCT, cone-beam computed tomography; CT, computed tomography; PET, positron emission tomography; IBSI, image biomarker standardization initiative; FFF, flattening-filter-free; MRIgRT, MRI-guided radiotherapy; ICC, intraclass correlation coefficient; CCC, concordance correlation coefficient; SNR, signal-to-noise-ratio; ROI, region of interest; NEX, number of excitations; NPC, nasopharyngeal carcinoma; GLCM, gray level co-occurrence matrix; GLRLM, gray level run length matrix; GLGM, gray level gradient matrix; SRE, short run emphasis; LRE, long run emphasis; RLNU, run length non-uniformity; RPC, run percentage; FOV, field of view; VOI, volume of interest.

## Appendix

**Table 6.** The Coefficient of Variation for the 130 Radiomics Features That Were Identified as Robust. Standard Deviation is Written Within Parenthesis.

Feature category	Feature	Liver	Kidney	Monthly	Daily
Shape-based	Long axis (mm,COM)	0.2 (0.09)	1.6 (0.61)	0.45 (0.16)	0.47 (0.01)
	Maximum 3D diameter (mm)	0.13 (0.07)	1.19 (0.5)	0.37 (0.14)	0.5 (0.09)
	V (voxels)	0.58 (0.23)	2.41 (0.87)	1.37 (0.99)	0.55 (0.18)
	Volume	0.58 (0.23)	2.41 (0.87)	1.37 (0.99)	0.55 (0.18)
	Surface area	0.73 (0.24)	1.64 (0.7)	0.58 (0.07)	0.52 (0.27)
	Surface-to-volume ratio	0.32 (0.09)	1.49 (0.54)	1.19 (1.05)	0.22 (0.1)
	Volume density (axis)	1.72 (0.89)	3.25 (1.15)	3.83 (3.43)	1.3 (0.93)
	Area density (axis)	1.23 (0.47)	1.8 (0.43)	1.51 (0.8)	0.95 (0.7)
	Volume density (convex)	0.58 (0.25)	0.79 (0.33)	1.02 (0.7)	0.5 (0.15)
	Area density (convex)	0.6 (0.27)	0.72 (0.37)	0.4 (0.23)	0.32 (0.08)
	Sphericity	0.42 (0.09)	1.04 (0.41)	0.8 (0.61)	0.2 (0.19)
	Asphericity	1.28 (0.28)	2.31 (0.87)	1.97 (1.53)	0.48 (0.47)
	Compactness 1	0.63 (0.14)	1.56 (0.62)	1.2 (0.91)	0.3 (0.29)
	Spherical disproportion	0.42 (0.09)	1.05 (0.41)	0.82 (0.64)	0.2 (0.2)
First order	Volume fraction at 0.10 intensity	0.45 (0.26)	0.57 (0.53)	2.88 (2.24)	0.3 (0.07)
	NIenergy	0.66 (0.35)	2.71 (0.98)	2.24 (0.61)	0.72 (0.26)
	Entropy	0.08 (0.03)	0.25 (0.09)	0.34 (0.06)	0.1 (0.04)
	Hist entropy	2.26 (1.37)	1.21 (0.47)	1.59 (0.65)	1.84 (0.7)
	Norm NIenergy	0.28 (0.23)	0.61 (0.35)	1.25 (0.71)	0.2 (0.08)
	Norm entropy	0.02 (0.02)	0.03 (0.02)	0.21 (0.11)	0.03 (0.01)
LoG sigma = 0.5	Energy	0.72 (0.37)	2.71 (0.93)	2.15 (0.72)	0.75 (0.26)
	Entropy	0.08 (0.04)	0.25 (0.09)	0.35 (0.08)	0.1 (0.04)
	Hist entropy	1.95 (0.51)	0.9 (0.47)	1.01 (0.25)	1.41 (0.18)
	Norm energy	0.36 (0.28)	0.65 (0.35)	1.97 (0.27)	0.46 (0.13)
	Norm entropy	0.02 (0.02)	0.03 (0.02)	0.27 (0.06)	0.05 (0.02)
LoG sigma = 1 mm	Entropy	0.21 (0.1)	0.29 (0.1)	0.5 (0.09)	0.58 (0.21)
	Hist entropy	2.01 (0.87)	1.85 (0.92)	2.63 (0.84)	3.45 (0.66)
	Norm entropy	0.15 (0.05)	0.06 (0.03)	0.34 (0.12)	0.49 (0.19)
LoG sigma = 1.5 mm	Coeff Vari	2.06 (1.66)	1.28 (0.58)	2.94 (0.8)	3.01 (0.58)
	Energy	1.88 (1.19)	2.92 (1.08)	3.01 (0.98)	3.01 (0.78)
	Entropy	0.21 (0.08)	0.29 (0.11)	0.39 (0.1)	0.35 (0.09)
	Hist entropy	1.89 (0.79)	1.79 (0.74)	1.93 (0.18)	2.17 (0.59)
	Norm energy	1.55 (1.31)	1.01 (0.46)	3.04 (1.42)	2.34 (0.54)
	Norm entropy	0.12 (0.09)	0.06 (0.02)	0.33 (0.13)	0.22 (0.05)
LoG sigma = 2 mm	Coeff Vari	3.03 (1.77)	1.68 (0.8)	2.3 (0.37)	3.71 (0.31)
	Energy	2.45 (1.49)	3.17 (1.06)	2.44 (0.8)	2.86 (0.58)
	Entropy	0.23 (0.12)	0.3 (0.11)	0.35 (0.16)	0.29 (0.04)
	Hist entropy	1.86 (0.7)	2 (0.9)	1.08 (0.09)	1.84 (0.34)
	Norm energy	2.31 (1.38)	1.36 (0.66)	2.12 (0.21)	2.96 (0.37)
	Norm entropy	0.17 (0.09)	0.08 (0.03)	0.22 (0.05)	0.29 (0.02)
LoG sigma = 2.5 mm	Coeff Vari	3.9 (2.04)	2.08 (1.12)	3.77 (0.86)	4.29 (0.12)
	Energy	3.22 (1.88)	3.45 (1.13)	3.86 (1.34)	3.83 (0.48)
	Entropy	0.3 (0.15)	0.31 (0.11)	0.53 (0.19)	0.46 (0.08)
	Hist entropy	2.2 (0.81)	2.02 (1.13)	1.44 (0.57)	2.33 (0.34)
	Norm energy	3.08 (1.77)	1.76 (0.98)	3.26 (0.63)	3.44 (0.07)
	Norm entropy	0.22 (0.13)	0.1 (0.04)	0.39 (0.12)	0.33 (0.03)
LoG sigma = 3 mm	Coeff Vari	4.33 (2.14)	2.1 (1.17)	4.28 (0.81)	3.41 (0.83)
	Energy	3.94 (2.4)	3.56 (1.33)	4.6 (1.58)	3.91 (0.27)
	Entropy	0.37 (0.21)	0.32 (0.13)	0.62 (0.22)	0.51 (0.04)
	Hist entropy	2.22 (0.84)	2 (1.03)	1.19 (0.31)	1.77 (0.11)
	Norm energy	3.52 (1.98)	1.82 (1.04)	3.72 (0.9)	2.72 (0.87)
	Norm entropy	0.27 (0.14)	0.1 (0.05)	0.45 (0.14)	0.27 (0.07)
Wavelet LLL	Coeff Vari	3.21 (1.21)	3.08 (1.39)	2.59 (0.9)	1.74 (0.62)
	Energy	1.41 (0.59)	3.06 (1.23)	2.08 (0.38)	1.33 (0.56)
	Entropy	0.14 (0.06)	0.28 (0.12)	0.27 (0.05)	0.16 (0.06)
	Hist entropy	1.27 (0.92)	1.12 (0.38)	1.3 (0.55)	0.95 (0.41)
	Norm energy	1.39 (0.53)	1.63 (0.7)	1.82 (0.72)	1.04 (0.39)
	Norm entropy	0.13 (0.04)	0.12 (0.06)	0.22 (0.09)	0.11 (0.04)
Wavelet LLH	Coeff Vari	3.7 (1.63)	4.55 (1.89)	3.34 (1.74)	2.62 (0.89)
	Entropy	0.69 (0.27)	1.13 (0.51)	0.78 (0.52)	0.37 (0.15)
	Hist entropy	1.15 (0.48)	1.4 (0.52)	0.49 (0.19)	0.6 (0.16)
	Norm entropy	0.7 (0.27)	1.13 (0.5)	0.79 (0.66)	0.4 (0.18)
Wavelet LHL	Entropy	1.41 (0.48)	0.99 (0.44)	1.17 (0.63)	0.78 (0.51)
	Hist entropy	1.12 (0.51)	1.62 (0.6)	0.49 (0.02)	0.54 (0.06)
	Norm entropy	1.4 (0.49)	0.96 (0.45)	1.11 (0.53)	0.76 (0.48)
Wavelet HLL	Entropy	1.37 (0.58)	1.16 (0.7)	1.24 (1.36)	0.53 (0.34)
	Hist entropy	1.19 (0.33)	1.24 (0.56)	0.59 (0.26)	0.66 (0.24)
	Norm entropy	1.38 (0.59)	1.09 (0.66)	1.21 (1.24)	0.56 (0.37)
Wavelet LHH	Entropy	2.13 (0.88)	2.28 (1.01)	3.24 (2.95)	1.49 (0.34)
	Hist entropy	1.15 (0.48)	1.46 (0.62)	0.41 (0.12)	0.46 (0.16)
	Norm entropy	2.13 (0.91)	2.24 (0.99)	3.26 (2.9)	1.51 (0.32)
Wavelet HLH	Entropy	2.27 (1.02)	3.07 (1.09)	3.09 (1.65)	3.24 (2.42)
	Hist entropy	0.95 (0.5)	2.13 (0.79)	0.52 (0.2)	0.58 (0.25)
	Norm entropy	2.26 (1.02)	3.08 (1.12)	3.04 (1.69)	3.22 (2.39)
Wavelet HHL	Entropy	1.55 (1.59)	2.98 (1.4)	4.99 (6.74)	0.83 (0.25)
	Hist entropy	1.13 (0.39)	1.22 (0.4)	0.57 (0.17)	0.54 (0.19)
Wavelet HHH	Coeff Vari	3.9 (2.14)	4.66 (2.59)	2.59 (1.13)	1.91 (1.26)
	Energy	0.57 (0.27)	2.64 (1.02)	1.88 (0.78)	0.8 (0.42)
	Entropy	0.07 (0.03)	0.24 (0.09)	0.24 (0.11)	0.1 (0.04)
	Hist entropy	1.23 (0.43)	1.18 (0.46)	0.56 (0.17)	0.37 (0.02)
	Norm energy	0.23 (0.15)	0.47 (0.28)	0.97 (0.55)	0.41 (0.27)
	Norm entropy	0.01 (0.01)	0.02 (0.01)	0.11 (0.06)	0.03 (0.02)
Laws EEE	Hist entropy	1.71 (0.77)	1.33 (0.65)	1.59 (0.63)	2.44 (0.69)
Laws EEL	Hist entropy	2.2 (1.09)	1.54 (0.77)	1.68 (0.38)	3.04 (0.78)
Laws EES	Hist entropy	1.56 (0.67)	1.23 (0.56)	2.4 (1.02)	2.92 (0.83)
Laws ELE	Hist entropy	1.83 (0.8)	1.45 (0.62)	1.71 (1.25)	2.93 (0.35)
Laws ELL	Hist entropy	2.16 (1.41)	1.41 (0.69)	1.69 (0.69)	1.98 (0.18)
Laws ELS	Hist entropy	1.84 (0.86)	1.69 (0.54)	1.38 (0.26)	2.63 (0.29)
Laws ESE	Hist entropy	1.52 (0.35)	1.4 (0.8)	1.79 (0.2)	2.81 (0.64)
Laws ESL	Hist entropy	1.42 (0.66)	1.97 (1.01)	2.48 (1.1)	3.01 (1.69)
Laws ESS	Hist entropy	1.59 (0.62)	1.29 (0.6)	2.55 (0.83)	2.59 (1.02)
Laws LEE	Hist entropy	1.86 (0.92)	1.82 (0.94)	1.58 (0.44)	0.83 (0.33)
Laws LEL	Hist entropy	1.81 (0.82)	1.82 (0.62)	1.24 (0.35)	1.21 (0.07)
Laws LES	Hist entropy	1.64 (0.81)	1.74 (0.96)	1.53 (0.21)	1.25 (0)
Laws LLE	Hist entropy	1.96 (0.87)	1.76 (0.68)	1.66 (0.73)	0.82 (0.1)
Laws LLL	Energy	0.66 (0.33)	2.69 (0.88)	2.2 (0.67)	0.7 (0.26)
	Entropy	0.08 (0.03)	0.25 (0.09)	0.3 (0.09)	0.1 (0.04)
	Hist entropy	2.71 (1.28)	1.32 (0.7)	0.82 (0.32)	2.15 (1.12)
	Norm energy	0.23 (0.24)	0.53 (0.35)	1.1 (0.81)	0.19 (0.06)
	Norm entropy	0.02 (0.02)	0.02 (0.02)	0.15 (0.1)	0.03 (0.01)
Laws LLS	Hist entropy	2.09 (1.03)	2.48 (1.17)	1.31 (0.46)	0.71 (0.34)
Laws LSE	Hist entropy	1.9 (0.7)	1.91 (1.11)	1.82 (0.68)	1.34 (0.03)
Laws LSL	Hist entropy	2.79 (1.41)	2.19 (0.82)	3.16 (1.12)	1.57 (0.39)
Laws LSS	Hist entropy	1.78 (0.68)	1.73 (0.76)	2.62 (1.02)	1.6 (0)
Laws SEE	Hist entropy	1.33 (0.72)	1.11 (0.35)	2.01 (0.38)	2.53 (0.19)
Laws SEL	Hist entropy	1.71 (0.89)	1.17 (0.55)	1.88 (0.34)	2.68 (0.31)
Laws SES	Hist entropy	1.63 (0.94)	1.47 (0.58)	2.26 (1.3)	2.94 (0.04)
Laws SLE	Hist entropy	1.76 (0.76)	1.44 (0.59)	1.4 (0.51)	3.08 (0.78)
Laws SLL	Hist entropy	2.49 (1.49)	1.66 (0.69)	1.79 (0.36)	2.49 (0.08)
Laws SLS	Hist entropy	1.61 (0.68)	1.33 (0.49)	2.46 (1.05)	3.35 (0.46)
Laws SSE	Hist entropy	1.38 (0.41)	1.51 (0.81)	2.57 (0.88)	3.32 (0.66)
Laws SSL	Hist entropy	1.23 (0.26)	1.98 (0.86)	2.66 (0.42)	3.15 (0.2)
Laws SSS	Hist entropy	1.7 (0.64)	1.49 (0.49)	2.75 (0.18)	3.56 (0.44)
GLCM	Entropy	3.46 (1.38)	1.72 (0.8)	1.59 (0.29)	2.13 (0.58)
	Mean	1.01 (0.48)	2.71 (0.85)	1.86 (0.91)	1.02 (0.1)
	Inverse difference moment	0.35 (0.13)	0.08 (0.04)	0.17 (0.1)	0.07 (0.03)
	Inverse difference	0.93 (0.37)	0.31 (0.15)	0.24 (0.07)	0.11 (0)
	Sum entropy	3.42 (1.39)	1.73 (0.8)	1.55 (0.52)	2.24 (0.38)
	Vnorm mean	0.7 (0.42)	0.63 (0.32)	0.96 (0.32)	0.77 (0.22)
	Gnorm entropy	3.46 (1.38)	1.72 (0.8)	1.59 (0.29)	2.13 (0.58)
	Gnorm sum entropy	3.42 (1.39)	1.73 (0.8)	1.55 (0.52)	2.24 (0.38)
	Gnorm mean	1.01 (0.48)	2.71 (0.85)	1.86 (0.91)	1.02 (0.1)
	VGnorm mean	0.7 (0.42)	0.63 (0.32)	0.96 (0.32)	0.77 (0.22)
GLRLM	Short-run emphasis	0.91 (0.59)	0.84 (0.45)	0.85 (0.24)	0.55 (0.16)
	Long-run emphasis	3.59 (2.32)	3.45 (1.81)	3.3 (1.57)	2.33 (1.01)
	Run length nonuniformity	3.58 (1.96)	4.17 (1.7)	4.6 (1.35)	3.18 (0.61)
	Run percentage	1.38 (0.66)	1.19 (0.62)	3.16 (1.25)	2.13 (0.25)
Fractal dimension	MeanLac1	3.81 (2.42)	4.36 (1.92)	2.34 (0.62)	0.68 (0.07)
	MeanLac2	1.1 (0.66)	1.25 (0.49)	1.85 (1.2)	0.92 (0.25)
	MeanLac3	0.6 (0.22)	2 (0.72)	1.06 (0.49)	0.78 (0.14)
